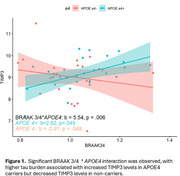# The Association Between Tau Burden and TIMP3 Varies by APOE4 Status

**DOI:** 10.1002/alz70856_107181

**Published:** 2026-01-08

**Authors:** Breanna M Holloway, Xin Wang, Ella T Lifset, Nadine C. Heyworth, Kitty K. Lui, Christian Harding, Emilie T. Reas, Atul Malhotra, Erin E. Sundermann, Sarah J Banks

**Affiliations:** ^1^ UC San Diego, La Jolla, CA, USA; ^2^ Department of Neurosciences, University of California, San Diego, La Jolla, CA, USA; ^3^ University of California, San Diego, La Jolla, CA, USA; ^4^ SDSU / UC San Diego Joint Doctoral Program in Clinical Psychology, San Diego, CA, USA; ^5^ UC San Diego Health, La Jolla, CA, USA

## Abstract

**Background:**

Tissue inhibitor of metalloproteinases‐3 (TIMP3) regulates extracellular matrix integrity, neuroinflammation, and blood‐brain barrier (BBB) function, all of which have bidirectional relationships with tau pathology in Alzheimer's disease (AD). Tau burden in medial temporal regions is a key marker of AD progression, yet its relationship with TIMP3 remains unclear. Given *APOE4*'s role in exacerbating tau pathology and BBB dysfunction, this study examines whether tau burden is associated with TIMP3 levels in older women with mild cognitive impairment (MCI) and whether *APOE4* moderates this relationship.

**Method:**

Data were collected from 50 older women (aged:72.4±4.2 years; 47.5% *APOE4* carriers) participating in the Women Inflammation and Tau Study at the University of California, San Diego. All women were at higher risk for AD, defined as a polygenic hazard score in the upper 50^th^ percentile and a score on the Telephone Montreal Cognitive Assessment suggestive of mild cognitive impairment (i.e., scores=13‐20/22). Tau burden was assessed using MK‐6240 standardized uptake value ratio from a composite region corresponding to BRAAK stages 3/4 and TIMP3 levels were measured in cerebrospinal fluid. Moderated linear regression was used to analyze tau burden in BRAAK 3/4 in relation to TIMP3 levels, and the moderating role of *APOE4*. The model adjusted for age and body mass index.

**Result:**

The overall model was statistically significant and explained 27% of the variance in TIMP3 levels, F(5, 44) = 2.43, *p* = .012. Results revealed a significant moderating effect of *APOE4* on the relationship between tau burden and TIMP3 levels, b = 5.54, *p* = .006. Specifically, greater tau burden was associated with higher TIMP3 levels, b=2.62, *p* = .049, among *APOE4* carriers; whereas greater tau burden was related to lower TIMP3 levels in non‐carriers, b = ‐2.91, *p* = .048.

**Conclusion:**

Our findings suggest a complex relationship between TIMP3 and tau pathology, potentially modulated by *APOE4* status. Specifically, higher tau burden was associated with increased TIMP3 levels in *APOE4* carriers but decreased TIMP3 levels in non‐carriers. Further research is warranted to clarify the mechanisms underlying TIMP3 regulation in AD, including its interactions with *APOE* genotype, tau pathology, and cerebrovascular integrity.